# Group standardization of Chinese experts specification of operating techniques for facial embedded thread lift

**DOI:** 10.3389/fsurg.2025.1750529

**Published:** 2026-01-16

**Authors:** Bing Shi

**Affiliations:** Chinese Association of Plastics and Aesthetics, Beijing, China

**Keywords:** Chinese expert consensus, facial anatomy, minimally invasive rejuvenation, PPDO threads, thread lift standardization

## Abstract

**Background:**

Despite the global popularity of minimally invasive thread lifts, the absence of standardized protocols has led to significant variations in outcomes. This study establishes China's first expert consensus (T/CAPA 009-2023) on facial thread lift techniques, addressing critical gaps in operator training, material selection, and anatomical precision.

**Methods:**

A multidisciplinary panel analyzed 2,143 PPDO thread procedures (2018–2022) across 35 institutions. The consensus framework integrates: 1) Graded facility/operator requirements (Grade III device management), 2) Anatomical stratification (SMAS, fat layers, ligament anchoring), and 3) Region-specific protocols (upper/mid/lower face, neck) with 14 illustrated surgical designs.

**Conclusion:**

Multicenter data and anatomical studies demonstrate that this consensus framework improves thread lift safety and efficacy, though further RCTs are warranted to confirm long-term outcomes The hierarchical protocol serves as a global benchmark for aesthetic training programs, particularly in Asian facial anatomy.

## 1 Introduction

Within past 10 years, facial embedded thread lift has acquired immense progress in China (1). Concerning its characteristics such as simplicity, minimally invasive, less downtime, low incidence of general anesthesia, conspicuous effects and low incidence of complications, which has been welcomed by beauty seekers (2). However, there are no relevant standards for facial embedded thread lift, as well as lack of strict standards for staffing qualification, operative methods, environmental facilities home and abroad. In order to strengthen the supervision and management of the industry and to standardize the operative process, we organized Chinese experts to formulate the Group standardization of operating techniques for facial embedded thread lift, for the sake of improving the safety and effectiveness of the operation and promoting the healthy and orderly development of the industry. The operative and designing methods in this standardization were summarized by experienced experts and clinicians. They contributed basic scheme for standardized operation to operators under the premise with full consideration of safety and effectiveness. Operators can adopt other diversified and personalized designing schemes according to the actual situation and personal experience, but they should follow the general principles of treatment in this standardization.

### 1.1 Specification of operating techniques for facial embedded thread lift

#### 1.1.1 Scope

This standardization specifies the treating principle, clinical indications, contraindications, requirements of medical institutions, qualifications of operators, classification and managing methods of thread, types and specifications of thread, burying levels, pre-treatment preparation, designing scheme, post-treatment management, prevention and treatment of major complications, etc. This article is applicable to the medical institutions which want to implement facial embedded thread lift.

#### 1.1.2 Normative references

There are no normative references in this article.

#### 1.1.3 Terminologies and definitions

There are no terminologies and definitions that need to be defined in this document.

#### 1.1.4 Principles of treatment

Thread lift achieves its function such as repositioning, elevating, tightening and constant sculpturing of facial tissue through direct and indirect anchoring of the deep temporal fascia and retaining ligaments, the hooking effect of barbed thread, and auxiliary effect of smooth thread and spiral thread (3–5). Further superimposed and sustaining effect will be achieved through fibrosis activated by tissue wrapping and sluggish absorbing of threads (6).

#### 1.1.5 Indications

a) Improving sagging of facial soft tissue and grooves
b) Facial beautification and shaping
c) Auxiliary increasing facial capacity
d) Combined application in facial rhytidectomy
e) Improving mild platysma and cervical skin laxity

The facial embedded thread lift can also improve fine lines and static wrinkles, skin color and skin texture to a certain extent (5, 7, 8). Problems such as severe laxity, grooves, wrinkles, over or less capacity, superficial pigmentation of the skin, were need to combined with other treatments (9–11).

#### 1.1.6 Contraindications

a) Pregnant and lactating women
b) Patients with malignant tumors and severe systemic diseases
c) Patients who are taking anticoagulant drugs
d) Patients with uncured mental illness

In addition, the satisfaction of the treatment decreased gradually with aging, which was negatively correlated with age (12). Absorbable thread should be the first choice for people over 50 years old, and combined therapy based on thread lift is more preferable (13).

#### 1.1.7 Requirements of medical institutions

a) It should be a legitimate medical plastic and cosmetic institution
b) Departments include at least, Cosmetic Surgery, Cosmetic Stomatology, Cosmetic Dermatology, Cosmetic Traditional Chinese Medicine, Cosmetic Ophthalmology
c) The operating environment should be an operating room or a sterilized treatment room, which should be equipped with necessary emergency equipment and medicines

#### 1.1.8 Qualification of operating physicians

The qualifications of operating physicians shall meet the relevant requirements of Article 11 of Chapter III of the Management Measures for Aesthetic Medicine Services, revised in 2016, issued by the National Health Commission of the People's Republic of China (NHC).
The physicians should receive formal training in facial embedded thread lift, and should complete at least more than 20 cases of facial embedded thread lift under the guidance of superior doctors.
Medical institutions in provinces, cities and administrative regions that have implemented the recording system of “cosmetology attending physicians” shall, on the basis of 8.1 and 8.2, be implemented in accordance with relevant provisions.
Certificated physicians with more than 3 years of aesthetic medicine experience can complete under the guidance of the above compliant physicians

#### 1.1.9 Classification, description and management of threads

##### 1.1.9.1 General requirement

The classification, application and management of thread should comply with the relevant provisions of the Medical Device Classification Catalogue of PRC.

##### 1.1.9.2 Classification

By April 1, 2022

Subdirectory 02, without origin of surgical instruments.
Grade I Product Category 13, surgical instruments and sewing instruments, materials.
Grade II Product Category 06, absorbable sutures.

Added on April 1, 2022

Subdirectory 13, without origin of surgical instruments.
Grade I Product Category 09, Plastic and General surgical implants.
Grade II Product Category 11, Plastic implant thread.

##### 1.1.9.3 Universal description

Thread material, usually composed of non-absorbable or absorbable polymers. Auxiliary tools, with or without a needle.

##### 1.1.9.4 Administrative measures

Abiding by supervision of Grade III medical device management.

#### 1.1.10 Types, specifications and characteristics of threads

##### 1.1.10.1 Facial embedded thread

###### 1.1.10.1.1 Non-barbed thread

The types of thread are divided into smooth thread and spiral thread with different diameters such as 3-0, 5-0 or 6-0. Thread length is between 50 mm to 90 mm. Cannula diameters are between 23G to 29G. Cannula has two kinds of needle, sharp one and blunt one. Material of thread is polydeoxy-cyclohexanone (PPDO). It is characterized by relatively simple operation, multiple embedding levels, which can play a variety of roles such as assisting fixation, volume augmentation and skin refreshing (14). In recent years, in addition to spiral threads, commonly available volumizing threads on the market also include cylindrical mesh types, broom types, twisted stick types, and other varieties.

###### 1.1.10.1.2 Barbed thread

The type of thread is U-shaped thread with bidirectional barb connects 17G needle. The diameter is 1-0, the length is 43 cm with 360°, 17 cm bidirectional spiral barb in each side. The material of thread is PPDO, which is characterized by its length and strong ability of pulling and hinging (15).

##### 1.1.10.2 Facial cone lift thread

The main body of thread is composed by polypropylene, the diameter is 3-0, the length is 400 mm, the cone diameter is controlled within 1.2–1.5 mm. The thread including 8 hollow polyl-lacto-glycolic acid (PLGA) cones in the same row, which are fixed by prefabricated thread junction clips with a spacing of 1 cm.

##### 1.1.10.3 Suturing thread

###### 1.1.10.3.1 Cannula one-way barbed thread

The length of thread is 45 cm–60 cm, the diameter is 2-0 or 3-0, and the form of the barb is akin to stamping flat fish bone. The caliber of cannula is 16G, the material is PPDO. It is characterized by long and firm linear body, relatively slow metabolic absorption under the same circumstances (16). The blunt and circular barbs provide benign cutting force to tissue, and the thread can be folded freely (17).

###### 1.1.10.3.2 Cannula bidirectional barbed thread

The length of thread is 80 mm–180 mm, the diameter is 0-0, 1-0 or 2-0. The formation of the barb can be punching and pressing simultaneously or can be cutting from main body of thread. The barbs are placed with bidirectional uniform space, which can be distributed 360° or flatly.

###### 1.1.10.3.3 Double straight needle two-way barbed thread

The length of thread is 80 mm–180 mm, the diameter is 2-0. The formation of the barb can be punching and pressing simultaneously or can be cutting from main body of thread. The distribution of barbs is 360° bidirectional arrangement, with smooth section in the middle. The material of thread is PPDO, with 10 cm/15 cm 21G three-sided cutting inverse angle needle. It is characterized by double needle connected with thread, flexible designed protocol, convenient operation (18). The angled design is equivalent to indirect anchoring without the need for knots (18).

#### 1.1.11 Selection of thread in different levels

##### 1.1.11.1 Subdermal or subcutaneous fat layer

Non barbed thread is preferable, for example, smooth thread or spiral thread.

##### 1.1.11.2 Interface between subcutaneous fat and SMAS

It is advisable to choose barbed thread, facial cone lift thread, suturing thread, etc.

##### 1.1.11.3 SMAS (platysma and palpebral part of lower palpebral orbicularis oculi muscle)

It is advisable to choose non barbed thread.

##### 1.1.11.4 Superperiosteal

It is advisable to choose non barbed thread, for example smooth thread and spiral thread.

#### 1.1.12 Pre-treatment preparation and po-treatment management

Attention should be paid to comprehensive systemic disease such as diabetes, hypertension, heart disease, hemorrhagic disease, chronic lung diseases, immune disorders, etc. in the collection of medical data (19, 20). Medical data concerning facial operation and injection need to include botulinum toxin injection, filler injection, face and neck liposuction, face and neck thread embedded treatment, etc. (21–24). Special attention should be paid to the history of application of anti-coagulant, and it is necessary to stop the drug for more than 2 weeks before facial embedded lift treatment (25).

Pre-treatment tests include blood routine, coagulation, virology test (hepatitis B, C, syphilis, AIDS, etc.) and electrocardiogram (4). Patients who need general anesthesia need to add tests such as liver and kidney function, blood sugar, urine routine, etc. (26). It is necessary to take standardized photos and spare skin before treatment.

The preparation of therapeutic instruments includes sterile treatment kits, dressings and corresponding sterile instruments, also, the sterilized requirements are strictly followed within the whole process (27). Post-treatment management includes keeping the face clean and dry, taking oral anti-swelling drug when necessary, limiting facial expression and excessive exercise for a short period after treatment, as well as avoiding heavy facial massage, etc. (5, 28, 29). According to the metabolic time of the thread, it is not appropriate to carry out the operation from instruments functioned as facial deep heating, tightening and lifting within a certain period after treatment (30).

#### 1.1.13 Anesthesia

Local or general anesthesia need to be chose concerning specific circumstance.

#### 1.1.14 Designing scheme of operation

**Special Note:** All methods were carried out in accordance with relevant guidelines and regulations. All experimental protocols were approved by the designated institution.

The core content of this research paper, “Chinese Expert Specification of Operating Techniques for Facial Embedded Thread Lift”, is an elaboration and report on an expert consensus and group standard (Standard No.: T/CAPA 009–2023). The development of such documents falls under methodological and guideline development studies. The process is based on existing publicly available literature, clinical evidence, and the collective experience and knowledge of multiple experts, aiming to establish standardized operational recommendations rather than constituting a prospective or retrospective clinical study involving direct interventions on patients.

The clinical data cited in the text to support the consensus viewpoints (e.g., the mentioned 2,143 procedures) were aggregated from routine clinical practices across participating expert institutions. These data were used solely to illustrate and demonstrate the feasibility and background of the technical specifications and were not collected or analyzed for the purpose of this study. According to internationally recognized scientific research ethics principles (such as the Declaration of Helsinki) and the review policies of most institutions, the development of expert consensus, guidelines, or technical standards is generally not defined as “research involving human subjects”. Therefore, the process of formulating this consensus did not require approval from an Institutional Review Board (IRB), and accordingly, obtaining informed consent from patients was also unnecessary.

##### 1.1.14.1 General principle

The pre-treatment design should be based on the assessment of facial sagging and aging, simultaneously, fully considered the general aesthetic requirements of Eastern peoples, such as elevating of outer corner of eyes, plump middle face, narrowed zygomatic arch and V-shaped face. Finally, adequate communication between patients and doctors is really essential.
Zoning specified designing scheme is more preferable, cross-zoning designing should not affect the normal activities of facial expression.
The proximal end of the barbed thread should be placed in a firm direct anchor point (31). In the running path, the stability of the thread can be enhanced for the sake of passing through the cutaneous branch of ligaments (31). The distal end should be precisely penetrated into the superficial fat septum to achieve the exact lifting and resetting effect (31).
Although absorbable thread such as PPDO can stimulate collagenesis to a certain extent and assist in increasing the volume, however, the effect is limited and cannot be replaced by the combined treatment of fillers (32). Therefore, it is advisable to avoid embedding unlimited number of smooth threads and spiral threads at various levels in order to achieve supporting and augmentation (33). The result of thread metabolism is scar, which will bring hidden dangers to subsequent treatments of facial diseases and cosmetic operation (34). Moreover, concentrated metabolism of the coating dyes for thread will induce color deposition in a short period of time, which will also bring short-term confusion to patients (35).

##### 1.1.14.2 Designing scheme of upper face operation

**Figure 1:**
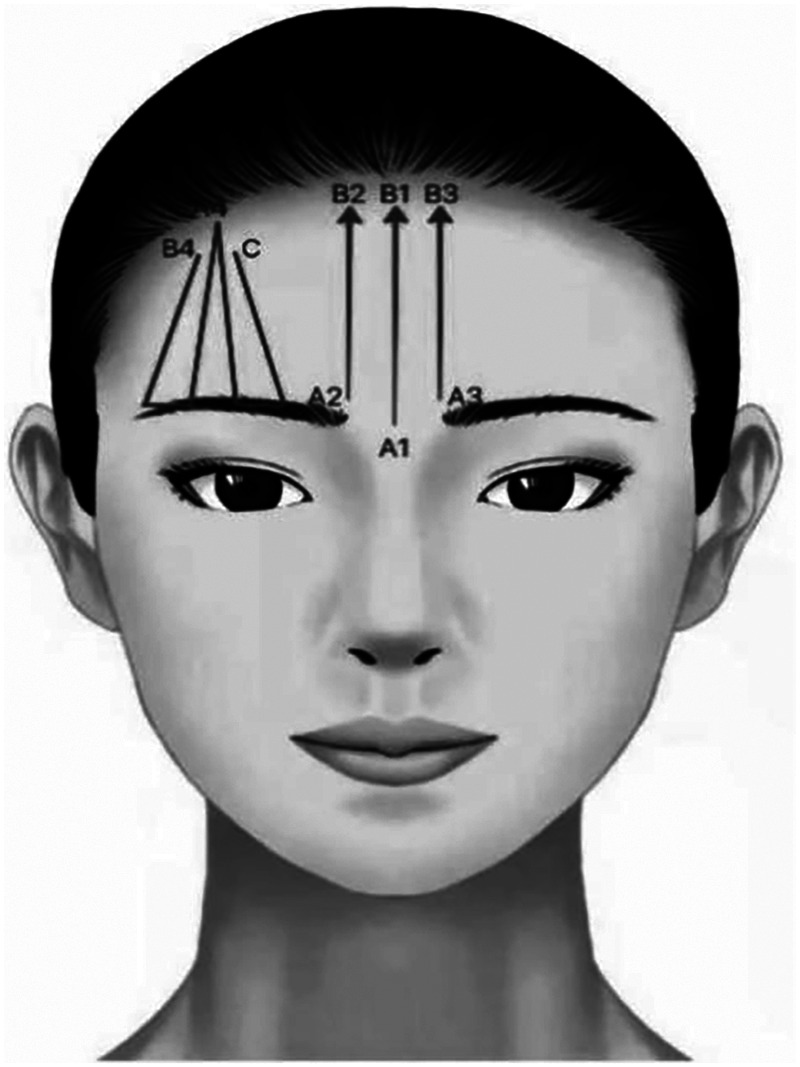
A4, B4, C: The left angled design is used to lift the eyebrow. The double straight needle two-way barbed thread is selected. The overall design can be based on the specific lifting position to determine whether it needs to shift in the direction of the brow or the tail of the eyebrow. It can also adjust the shape of eyebrow. A1–B1, A2–B2, A3–B3: The middle part is designed to lift the head of eyebrow, improve the sagging and partially improve the sunken of mid-forehead. It is advisable to choose cannula bidirectional barbed thread.

**Characteristic**. The left angled design is used to lift the eyebrow. The double straight needle two-way barbed thread is selected. The overall design can be based on the specific lifting position to determine whether it needs to shift in the direction of the brow or the tail of the eyebrow. It can also adjust the shape of eyebrow. The middle part is designed to lift the head of eyebrow, improve the sagging and partially improve the sunken of mid-forehead. It is advisable to choose cannula bidirectional barbed thread (Figure 1).

##### 1.1.14.3 Designing scheme of middle face

**Figure 2:**
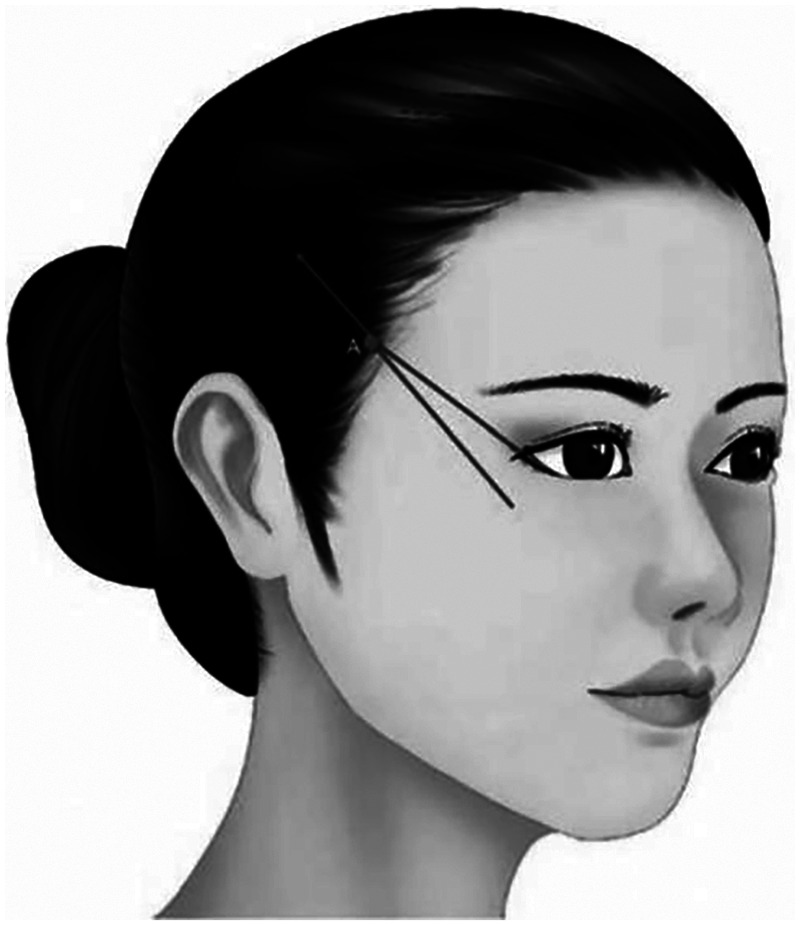
Cannula bidirectional barbed thread was selected. The upper part was used to lift the ocular tail by anchoring the lateral orbital thickening, and the lower part was used to lift the superolateral part of the zygomatic fat pad to improve the mild orbitozygomatic sulcus and sagging of inferior eyelid. Knotting and reversely anchored to the deep temporal fascia.

**Characteristic**. Cannula bidirectional barbed thread was selected. The upper part was used to lift the ocular tail by anchoring the lateral orbital thickening, and the lower part was used to lift the superolateral part of the zygomatic fat pad to improve the mild malar-palpebral groove and sagging of inferior eyelid. knotting and reversely anchored to the deep temporal fascia (Figure 2).

**Figure 3:**
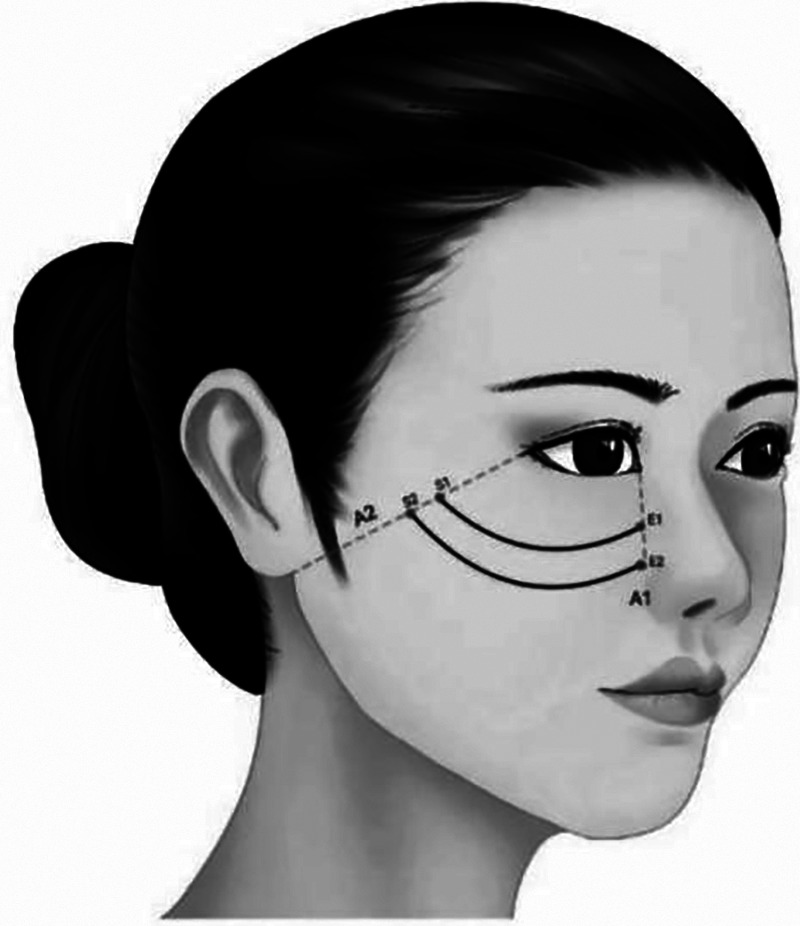
The cannula bidirectional barbed thread or double straight needle two-way barbed thread was used to lift the zygomatic fat pad, while taking into account the fullness of the middle face and completing the aesthetic plane. (S1–E1, S2–E2).

**Figure 4:**
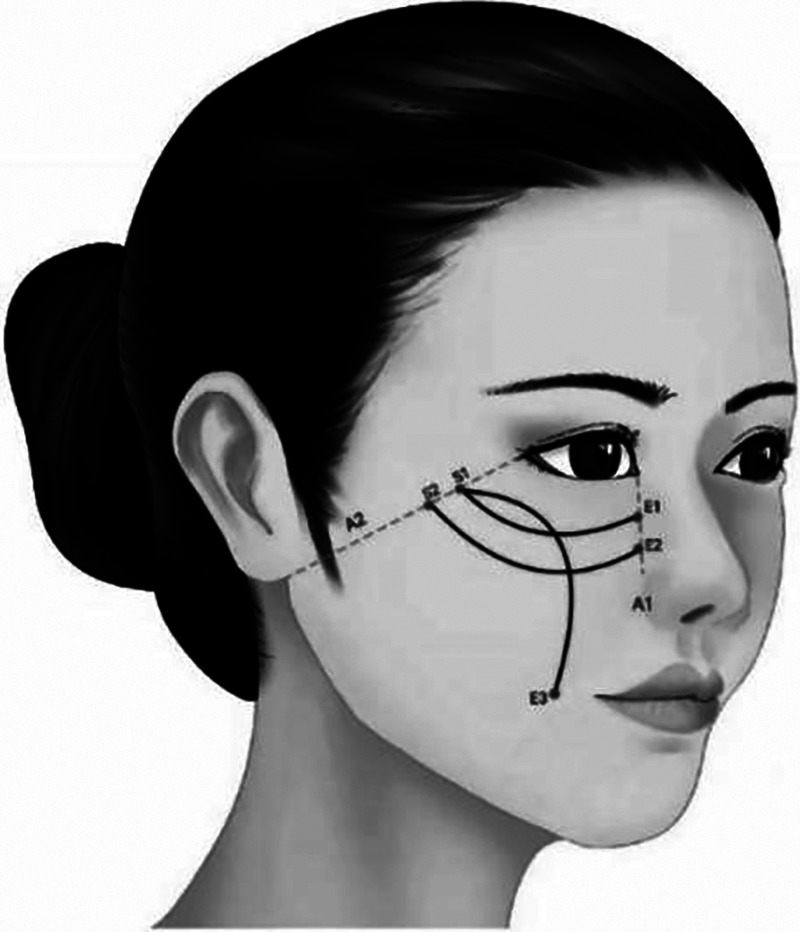
Adds an adducent fixation design on the basis of Figure 3, which is more suitable for those with mild zygomatic arch enlargement (S1–E3).

**Characteristic**. The cannula bidirectional barbed thread or double straight needle two-way barbed thread was used to lift the zygomatic fat pad, while taking into account the fullness of the middle face and completing the aesthetic plane. Figure 3 adds an adducent fixation design on the basis of Figure 4, which is more suitable for those with mild zygomatic arch enlargement (Figures 3, 4).

**Figure 5:**
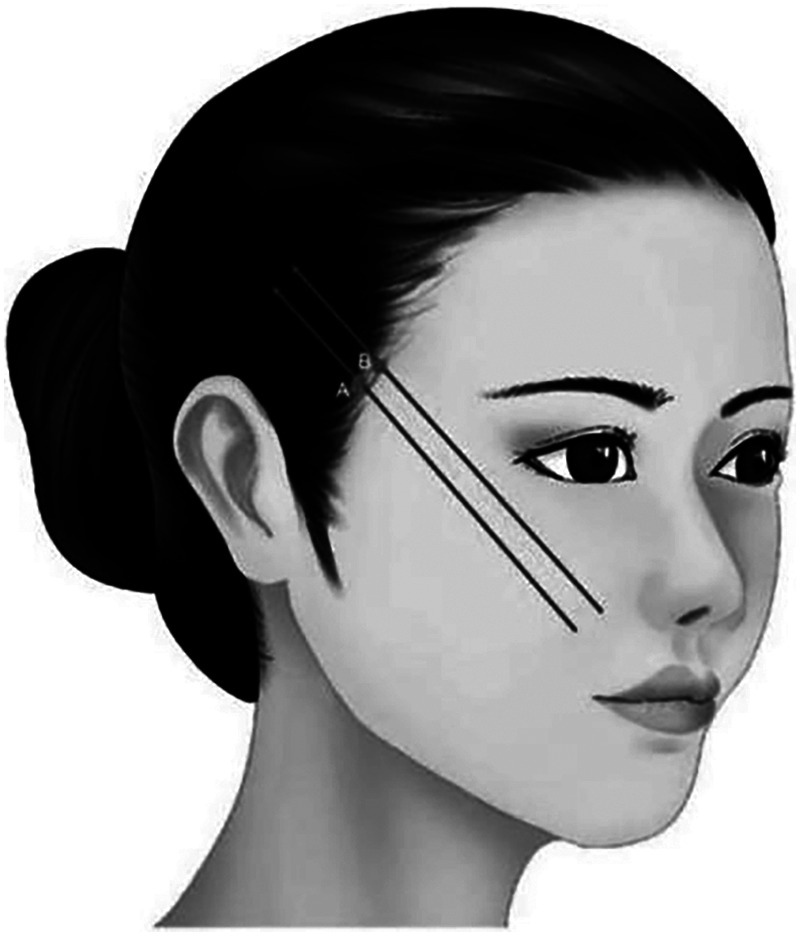
Cannula bidirectional or unidirectional barbed thread is used to lift the zygomatic fat pad superolateral to improve the nasolabial groove, especially suitable for the patients with mild zygomatic arch enlargement. The proximal end of the threads placed in the nasolabial fat septum, and the distal end can be knotted or not, but all of them need to be reversely anchored to the deep temporal fascia.

**Characteristic**. Cannula bidirectional or unidirectional barbed thread is used to lift the zygomatic fat pad superolateral to improve the nasolabial groove, especially suitable for the patients with mild zygomatic arch enlargement. The proximal end of the threads placed in the nasolabial fat septum, and the distal end can be knoted or not, but all of them need to be reversely anchored to the deep temporal fascia (Figure 5).

##### 1.1.14.4 Designing scheme of lower face

**Figure 6:**
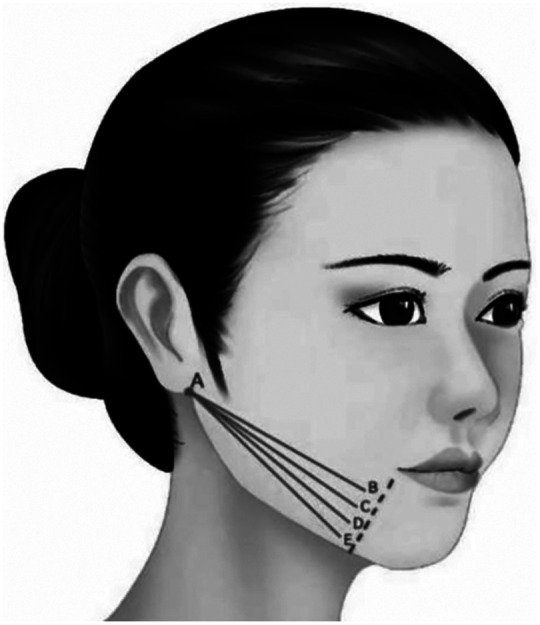
Cannula bidirectional or unidirectional barbed thread or facial cone elevated thread is mainly used to improve the jowl and indirectly reshape the mandibular margin through indirect anchoring technique of platysma auricular ligament. The proximal thread should be precisely embedded in the mandibular fat septum (A–B, A–C, A–D, A–E).

**Characteristic**. Cannula bidirectional or unidirectional barbed thread or facial cone elevated thread is mainly used to improve the jowl and indirectly reshape the mandibular margin through indirect anchoring technique of platysma auricular ligament. The proximal thread should be precisely embedded in the Jowl fat (Figure 6).

**Figure 7:**
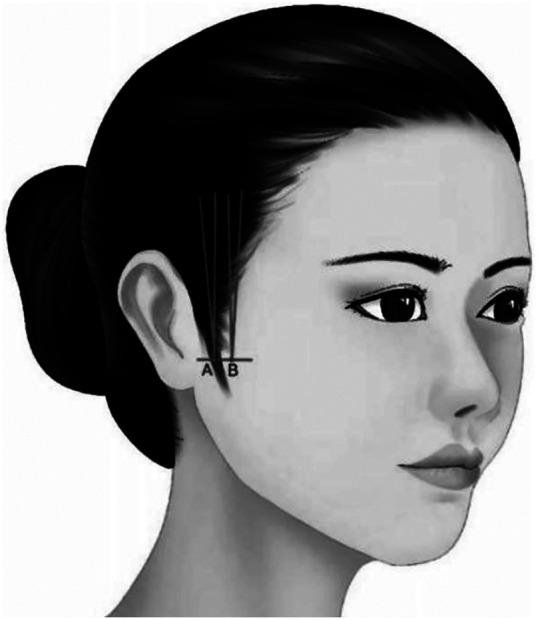
Reversing design was made on the upper margin of zygomatic arch by using the cannula bidirectional barbed thread. The proximal of the thread could be knotted or not, and the distal end is anchored in the deep temporal fascia. It is mainly used to improve the lateral buccal sagging and assist in reshaping the morphology of mandibular margin.

**Characteristic**. Reversing design was made on the upper margin of zygomatic arch by using the cannula bidirectional barbed thread. The proximal of the thread could be knoted or not, and the distal end is anchored in the deep temporal fascia. It is mainly used to improve the lateral buccal sagging and assist in reshaping the morphology of mandibular margin (Figure 7).

**Figure 8:**
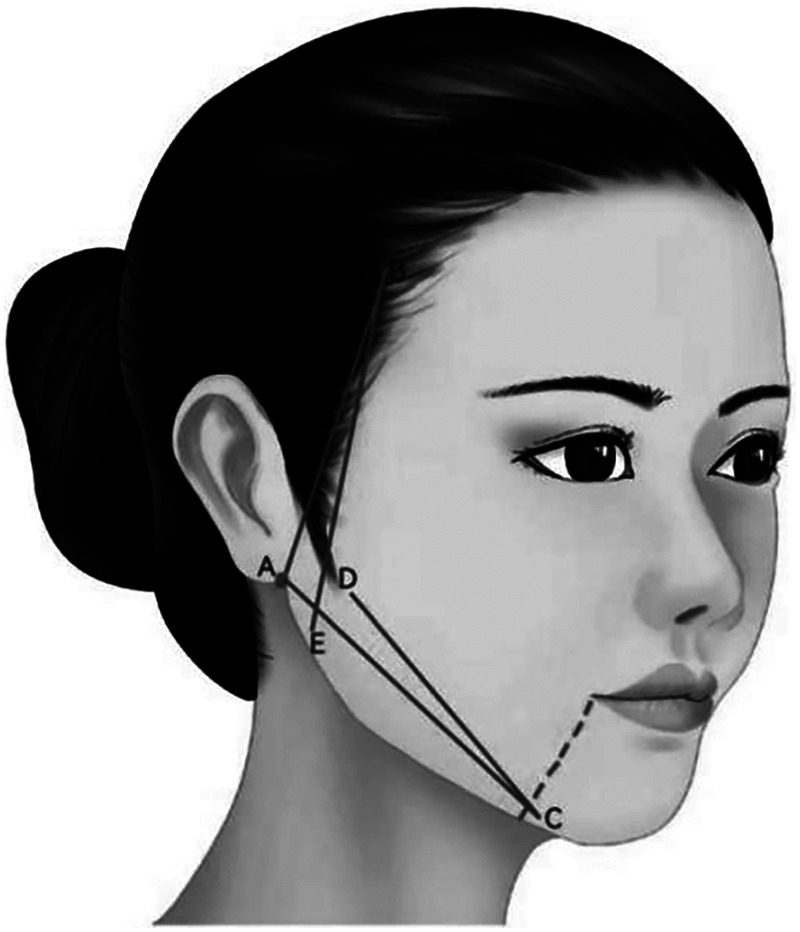
The integrated design of mandibular margin and lateral buccal is carried out by burying the double straight needle two-way barbed thread in the platysma auricular ligament. It is mainly used to improve the lateral buccal sagging and reshaping the morphology of mandibular margin (A–B, A–C, B–E, C–D).

**Characteristic.** The integrated design of mandibular margin and lateral buccal is carried out by burying the double straight needle two-way barbed thread in the platysma auricular ligament. It is mainly used to improve the lateral buccal sagging and reshaping the morphology of mandibular margin (Figure 8).

##### 1.1.14.5 Designing scheme of middle lower face

**Figure 9:**
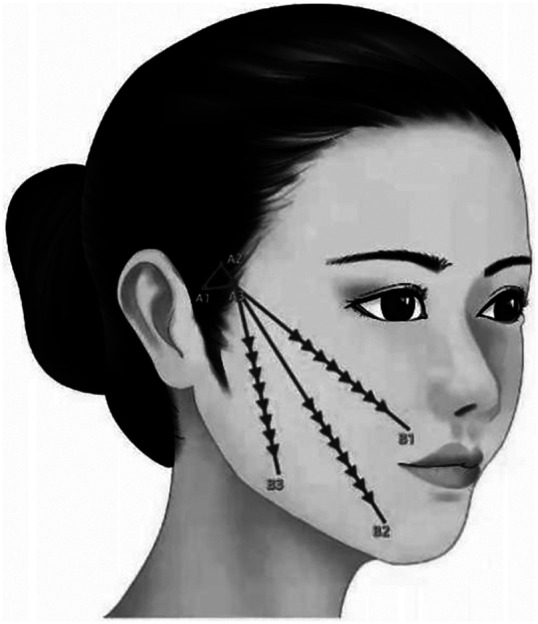
This group of designs used to improve mild to moderate sagging of middle lower face, deepening of nasolabial groove, jowl and morphology of mandibular margin. A variety of threads can be chose to finish the designs respectively. For example, anchoring thread design of cone elevated thread. The distal of threads should be anchored in the deep temporal fascia, and the proximal of threads should be precisely embedded in the nasolabial fat septum.

**Figure 10:**
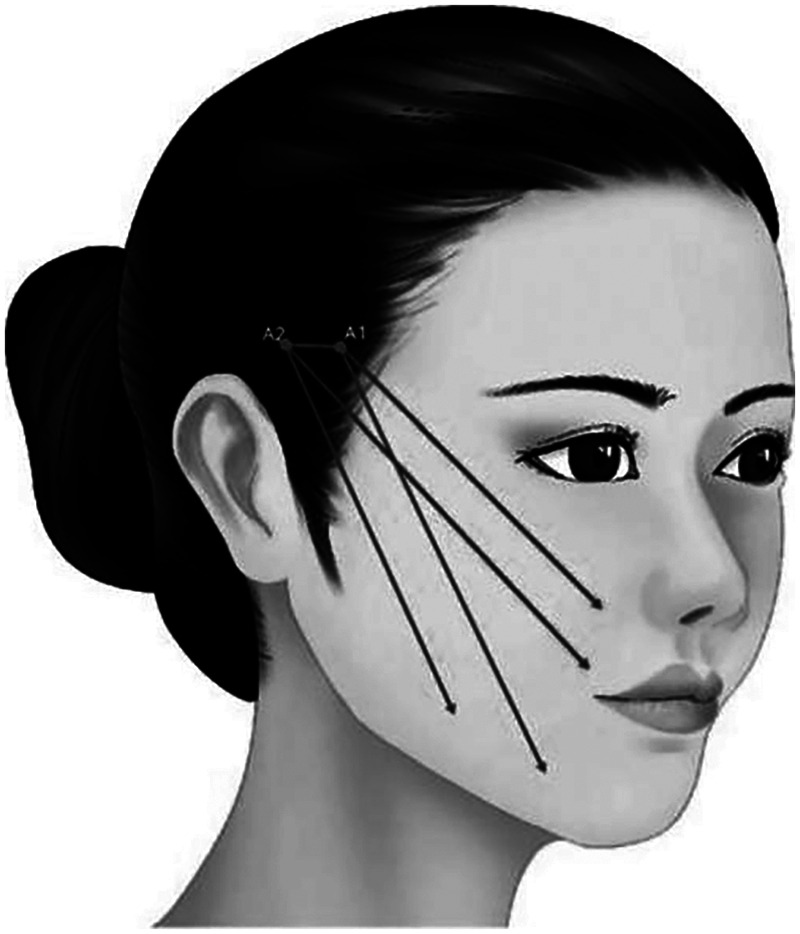
This group of designs used to improve mild to moderate sagging of middle lower face, deepening of nasolabial groove, jowl and morphology of mandibular margin. A variety of threads can be chose to finish the designs respectively. For example, inverted U design of bidirectional barbed thread. The distal of threads should be anchored in the deep temporal fascia, and the proximal of threads should be precisely embedded in the nasolabial fat septum.

**Figure 11:**
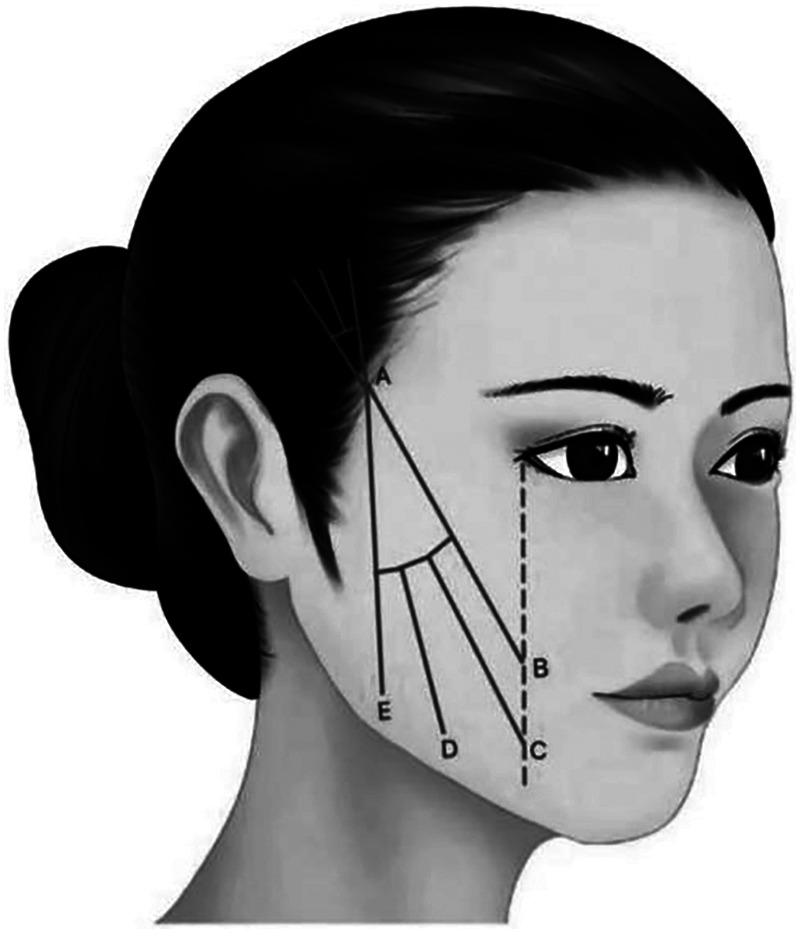
This group of designs used to improve mild to moderate sagging of middle lower face, deepening of nasolabial groove, jowl and morphology of mandibular margin. A variety of threads can be chose to finish the designs respectively. For example, folding and fixing forward and backward design of cannula unidirectional or bidirectional barbed thread, etc.

**Figure 12:**
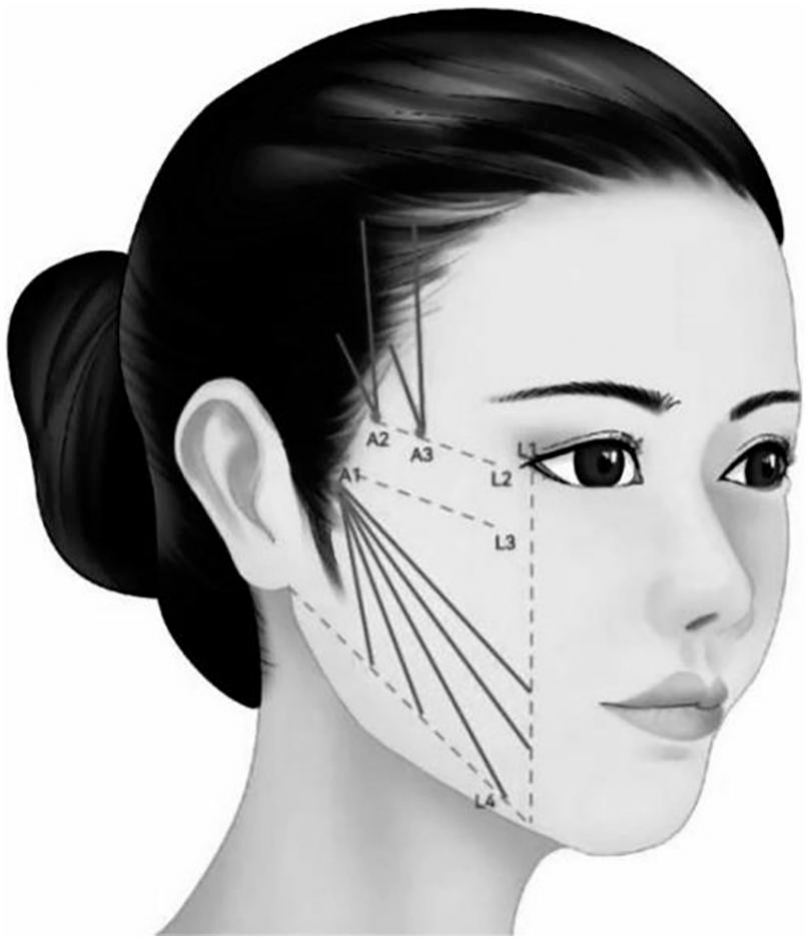
This group of designs used to improve mild to moderate sagging of middle lower face, deepening of nasolabial groove, jowl and morphology of mandibular margin. A variety of threads can be chose to finish the designs respectively. For patients with obvious external enlargement of zygomatic arch, cannula bidirectional barbed thread can be applied into forward and backward design of upper or lower edge of zygomatic arch respectively.

**Characteristic**. This group of designs used to improve mild to moderate sagging of middle lower face, deepening of nasolabial groove, jowl and morphology of mandibular margin. A variety of threads can be chosen to finish the designs respectively. For example, anchoring thread design of cone elevated thread (Figure 9), inverted U design of bidirectional barbed thread (Figure 10), folding and fixing forward and backward design of cannula unidirectional or bidirectional barbed thread, etc. (Figure 11). For patients with obvious external enlargement of zygomatic arch, cannula bidirectional barbed thread can be applied into forward and backward design of upper or lower edge of zygomatic arch respectively (Figure 12). The distal of threads should be anchored in the deep temporal fascia, and the proximal of threads should be precisely embedded in the nasolabial fat septum (Figures 9, 10).

##### 1.1.14.6 Designing scheme of buccal and cervical zone

**Figure 13:**
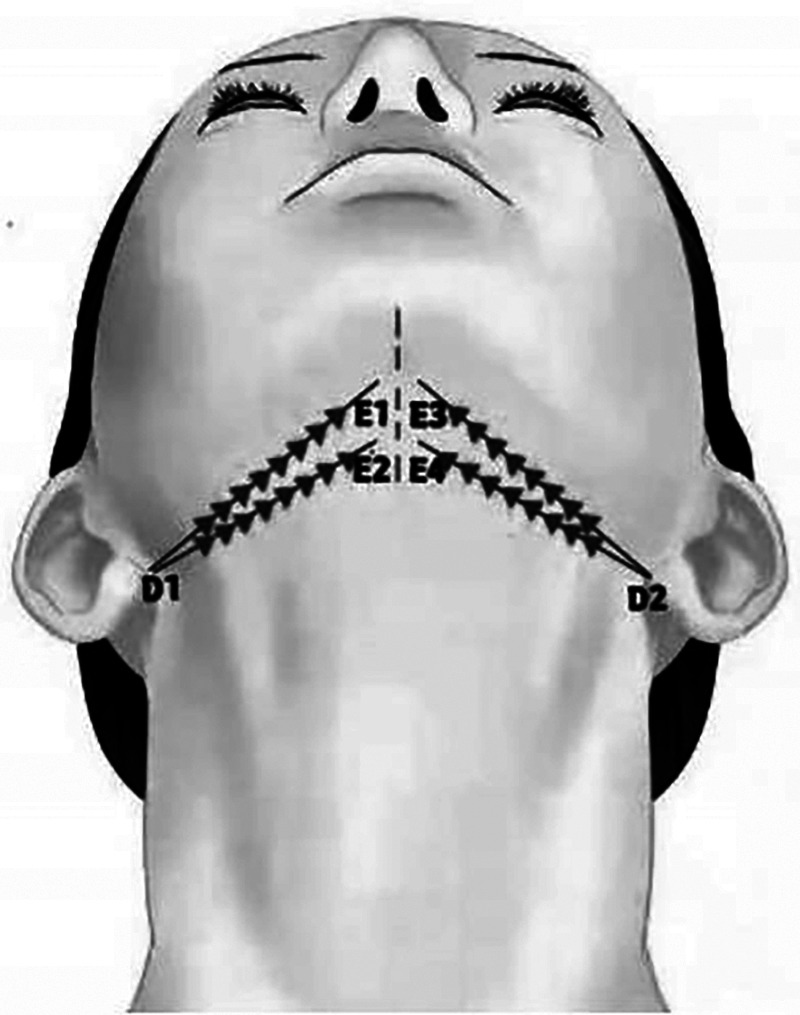
This group of designs used to improve mild to moderate cervical sagging and morphology mentocervical angle. A variety of threads can be chose to finish the designs respectively. For example, anchoring thread design of cone elevated thread. Make full use of platysma auricular ligaments, both sides of threads are all indirectly anchored.

**Figure 14:**
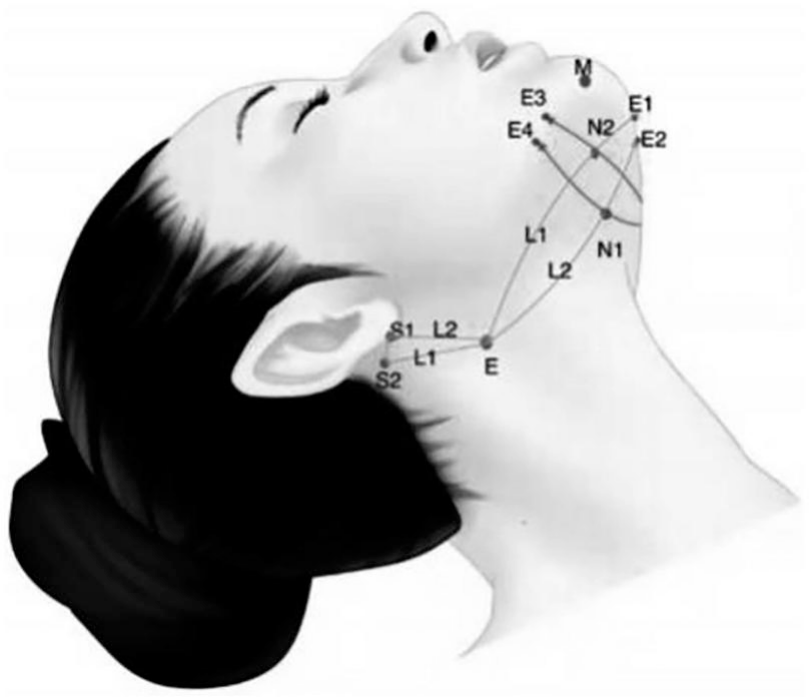
This group of designs used to improve mild to moderate cervical sagging and morphology mentocervical angle. A variety of threads can be chose to finish the designs respectively. For example, burying design of bidirectional barbed thread. Make full use of platysma auricular ligaments, both sides of threads are all indirectly anchored.

**Figure 15:**
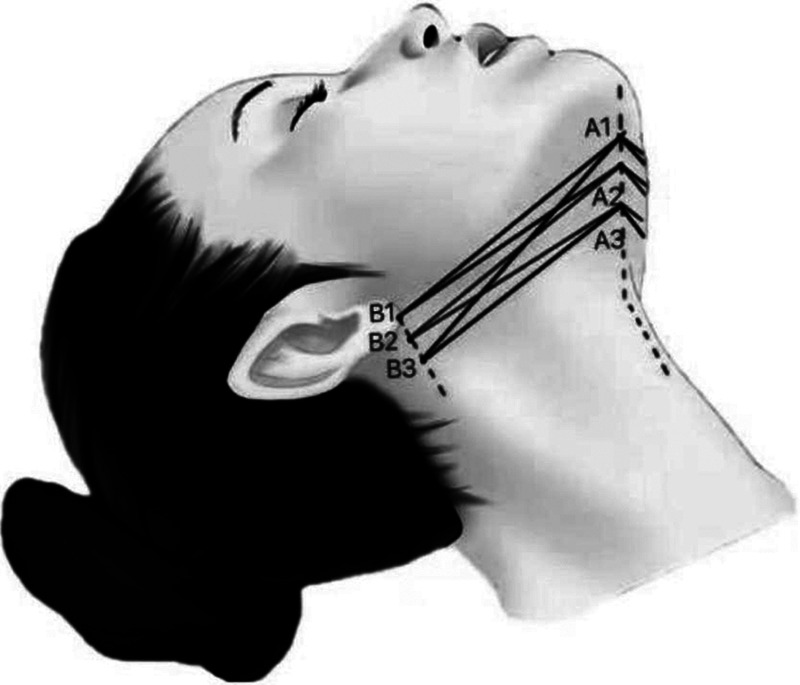
This group of designs used to improve mild to moderate cervical sagging and morphology mentocervical angle. A variety of threads can be chose to finish the designs respectively. For example, net design of double straight needle thread. Make full use of platysma auricular ligaments, both sides of threads are all indirectly anchored.

**Characteristic**. This group of designs used to improve mild to moderate cervical sagging and morphology of mentocervical angle. A variety of threads can be chosen to finish the designs respectively. For example, anchoring thread design of cone elevated thread (Figure 13), burying design of bidirectional barbed thread (Figure 14), net design of double straight needle thread (Figure 15). Make full use of platysma auricular ligaments, both sides of threads are all indirectly anchored.

##### 1.1.14.7 Designing scheme of smooth thread and spiral thread in buccal, neck and face

**Characteristic**. Smooth thread and spiral thread can be applied to different designs according to different requirements. Application of crossroad net design can boost the volume. Combined application of spiral thread and barbed thread through vertical burying can assist the lifting effect of barbed thread. The spiral thread buries perpendicularly to the frontal muscle, platysma and other intramuscular burying can relax the muscle respectively. The operation above can also facilitate the improvement of frontal and neck lines as well as lifting of lower face. The intramuscular thread parallel to the orbicularis oculi muscle can enhance the muscular strength to improve the herniation of lower eyelid fat (Supplementary Figures S1, S2).

#### 1.1.15 Common complications and prevention

##### 1.1.15.1 Local swelling and bruising

It is usually caused by local trauma and impairments derived from repeated puncture (36). Preventive approaches include add epinephrine into local anesthetics which can reduce hemorrhage, be familiar with local anatomic structure, make sure accurate puncture in operation to minimize trauma (37). Treatments mainly include hot compress, physiotherapy and application of specific medication (38).

##### 1.1.15.2 Hematoma

It is usually caused by damaging of large and middle arteriovenous systems (39). Preventive approaches include familiarizing physicians with the atlas of blood vessels and taking care of operational level (40). Treatments include aspiration, compression, drainage, and hematoma removal if necessary (41).

##### 1.1.15.3 Mild discomforts include local tightness, stiff facial expression, tingling, etc.

Generally, it is caused by the tightening and enhancing effect after burying the thread immediately, and it is also related to the cross-zone treatment (42). The stinging pain is caused by slight displacement and slippage of the serration, irritation of the subcutaneous sensory nerve (43). Symptoms mentioned above are generally limited to one week after embedded (44). Preventive approaches include avoiding massages in the short term and reducing large facial movements (43). The symptoms can be recovered without treatment naturally (43).

##### 1.1.15.4 Uneven and local obvious depression after treatment

It is usually caused by shallow burying of thread or superficial puncture of facial retaining ligament (44). It is most likely to occur in three regions, the region of middle buccal sulcus, the region of junction of zygomatic fat pad and masseteric cutaneous ligament as well as the region of the zygomatic arch ligament and parotid masseteric fascial ligament (45). Preventive approaches include fully evaluate the subcutaneous fat thickness of patients, clearly define and mark ligament-related anatomy, burying level of thread need to be accurate within operation (10). The symptoms can be treated according to specific situation, mild case can be treated by local massage or restored within a month automatically (46). For patients who cannot waiting or the depression is really severe, combined treatment with fillers or subcision near the depressed site to loosen the adhesion are applicable (47).

##### 1.1.15.5 Bilateral asymmetry

It is mainly caused by asymmetry of bilateral designing marks, uneven dosage of local anesthetics, inaccurate puncturing level and difference concerning amount and direction of buried thread (48). The consistency of facial symmetry and sagging should be evaluated correctly before treatment (48). Symmetrical concept and precise operation are consistent in whole process, comparison and adjustment will always stay in tuned. Mild asymmetry can recover naturally within 1 month (7). Sever asymmetry can be corrected by thread or filler.

##### 1.1.15.6 Tendering and exposing of thread residue

The main reason is that the puncturing points at both ends of the thread are shallow and close to or touching the dermis (49). With the movement of face and the effect of gravity, the thread gradually pushes up the skin, causing pain or breaking the skin (50). In addition, another reason is due to excessive facial movement to break the thread (51). Preventive method is pay attention to burying depth of both ends of the thread (52). Cutting off the excess thread directly or indirectly if it happened (52).

##### 1.1.15.7 Local crossing protrusion of threads

Crossing of barbed threads is easy to noose and causes bumps, stinging, etc. (8). In principle, the barbed threads should not be buried intersectly (8). Or at least, the cross-designed threads need to be placed in different depth. Making a small incision at the puncturing point or cutting the thread residue as short as possible can also help to avoid the symptom (53).

##### 1.1.15.8 Infections after treatment

Main reasons are as follows.

a) Infection related high risk factors such as immune system disease, history of hormone application and diabetes, or impaired glucose tolerance were not recorded before treatment.
b) Aseptic principle was not strictly followed in the treatment.

After infection, systemic medication is the main method in the early stage (54). When abscess occurs, local puncture and drainage can be used (54). The infected site and the thread need to be removed (55). If necessary, secretion should be sent for bacterial culture to guard against the possibility of non-tuberculous mycobacterium infection.

##### 1.1.15.9 Neural damage

The main reason is that the concentration of local anesthetic is too high, the quantity is too large and the waiting time is too long (56). It is also possible that the neural damage derived from improper depth of injection or burying (56). Damage is generally reversible and can recover naturally. Open surgical repair should be considered to treat permanent neural injury if the patient fails to recover after 2–3 months (57).

##### 1.1.15.10 Injury of parotid gland and duct

Due to improper puncture during treatment, it can enter the capsule of parotid gland or essence of parotid gland, which will lead to painful, local swelling (58). The symptoms will be aggravated when eating. Preventive methods include be familiar with the corresponding local anatomy and do not puncture too deeply (59). The therapy is mainly conservative treatment with local pressure, the symptoms can be relieved within a week. If further local intractable swelling occurs, it may cause by leakage of gland attributed to parotid duct injury, which requires emergent specialist treatment (60).

#### 1.1.16 Recommendations for combined therapy

The combined application of different specifications of threads in different areas of the face can achieve better immediate and long-term outcomes through domino effect (61).
Injection of botulinum toxin into the frontal muscle at multiple points can improve the frontal wrinkle more significantly due to muscle relaxation, so as to reduce the exposure of the thread caused by muscle extrusion (62). Injection of botulinum toxin into the platysma to reduce the antagonistic force from its downward contraction, so that the effect of thread lifting is more remarkable (63).
The combined application of soft tissue fillers can further correct the volume loss, further improve the lacrimal groove, buccal groove and alleviate the dark circles (64–66). Precise ligament supporting and reparative deep injection can achieve the long-term effect of synergistic treatment (13).
The combined application of mesotherapy can achieve the therapeutic effects such as repairing skin barrier, improving skin color spots, narrowing pores and weakening color spots (3, 67, 68).
The combined application of acousto-optic instruments can produce the synergistic effect of tightening and lifting, and can also significantly improve superficial color spots and pigmentation (69).

## Discussion

This expert consensus introduces China's inaugural standardized protocol for facial thread lifting (T/CAPA 009-2023), which systematically addresses significant deficiencies in practitioner qualification, anatomical targeting, and material standardization. By integrating a tiered framework encompassing facility classification, layered anatomical strategies (including SMAS, adipose tissue, and ligament-based fixation), and area-specific technical plans, this protocol has demonstrated enhanced safety and efficacy across 2,143 documented PPDO thread procedures. Notably, comparative analysis indicates that consistent application of this protocol is associated with a marked reduction in major complications—such as parotid gland injury and thread exposure—relative to non-standardized approaches. This observation aligns with the global shift toward anatomically precise methodologies in minimally invasive rejuvenation (4, 25).

While prior studies focused on thread mechanics or isolated techniques, this consensus integrates multidisciplinary expertise (plastic surgery, dermatology, TCM) to optimize outcomes for Asian facial anatomy (4, 17, 31, 45). For example, our zygomatic arch anchoring technique (Figure 7) minimizes thread migration risks—a common limitation in Caucasian-centric protocols (34). The emphasis on indirect anchoring via deep temporal fascia (Section 14.1.3) corroborates recent biomechanical studies, yet diverges from Western “direct suspension” approaches (40, 45, 46).

This protocol establishes a scalable framework through its graded operator certification (Section 8) and metabolic risk stratification for threads (Section 14.1.4), offering a transferable model for developing aesthetic markets. A recognized limitation, consistent with recent meta-analyses, is the absence of randomized controlled trials directly comparing thread materials such as PPDO and PLGA (27, 33).

The core innovation of this consensus is its holistic synthesis of procedural standardization, anatomical accuracy, and reproducible technique—transcending fragmented technical descriptions. As China's first national standard in this field, it systematically connects tiered operator credentialing with stratified anatomical planning and a complete library of 14 site-specific designs. Importantly, it introduces key advances including indirect anchoring through the deep temporal fascia and clear metabolic risk stratification—novel elements tailored to Asian facial morphology and long-term safety. This structured framework elevates thread lifting from a variable craft to a standardized, safer clinical discipline.

## Limitations

First, the data predominantly come from Chinese populations, and the generalizability of findings to ethnic groups with distinct facial anatomical characteristics (e.g., subcutaneous fat thickness, ligamentous structures) requires further validation. Second, with a median follow-up of 12 months—sufficient for assessing short-term complications—the evaluation of long-term outcomes such as thread resorption kinetics and sustained collagen remodeling (typically requiring 24–36 months) remains limited. Furthermore, despite adherence to Grade III medical device management, heterogeneity in thread materials and specifications across manufacturers may introduce variability in clinical outcomes. Lastly, aesthetic assessments (e.g., “Eastern aesthetic preferences”) involve subjective elements; future studies should incorporate objective tools such as 3D photogrammetry and ultrasound imaging to quantify outcomes, and prospective research is needed to further verify the clinical efficacy and broad applicability of these guidelines.

## Conclusion

In summary, this expert consensus represents a major step toward standardizing facial thread lift techniques by integrating anatomical precision with clinical applicability. The framework introduces evidence-based guidelines for practitioner training, material selection, and site-specific designs, which collectively contribute to lowering complication rates while respecting Asian aesthetic ideals. Key technical contributions—such as indirect deep temporal fascia anchoring and metabolic risk stratification—directly tackle issues of thread migration and durability. Beyond its immediate clinical relevance, this work may stimulate wider adoption of standardized practices in minimally invasive rejuvenation. We therefore propose three forward-looking initiatives: (1) cross-ethnic validation through international collaboration, (2) longitudinal outcome studies exceeding 24 months, and (3) development of affordable adaptations for resource-limited settings. Pursuing these directions will help realize the full promise of thread lifting as a safe, effective, and universally applicable approach to facial rejuvenation.

## Data Availability

The raw data supporting the conclusions of this article will be made available by the authors, without undue reservation.

## Supplementary material

Supplementary Figures S1 Smooth thread and spiral thread can be applied to different designs according to different requirements. Application of crossroad net design can boost the volume. Combined application of spiral thread and barbed thread through vertical burying can assist the lifting effect of barbed thread. The spiral thread buries perpendicularly to the frontal muscle, platysma and other intramuscular burying can relax the muscle respectively. The intramuscular thread parallel to the orbicularis oculi muscle can enhance the muscular strength to improve the herniation of lower eyelid fat.

Supplementary Figures S2 The operation can also facilitate the improvement of neck lines as well as lifting of lower face.
