# Correction: Group standardization of Chinese experts specification of operating techniques for facial embedded thread lift

**DOI:** 10.3389/fsurg.2026.1793029

**Published:** 2026-02-05

**Authors:** Bing Shi

**Affiliations:** Chinese Association of Plastics and Aesthetics, Beijing, China

**Keywords:** Chinese expert consensus, facial anatomy, minimally invasive rejuvenation, PPDO threads, thread lift standardization

Supplemental material **Supplementary file 1** was omitted. The list of “Participants in standard development” was inadvertently omitted from the original submission. This file acknowledges all experts who contributed to the consensus-building process described in the study, which forms the basis of the expert specification (Standard No.: T/CAPA 009-2023). The file(s) have now been published.

The original version of this article has been updated.

