# Supplementary material for: Group standardization of Chinese experts specification of operating techniques for facial embedded thread lift
Source: Front Surg. 2026 Jan 16;12:1750529. doi: 10.3389/fsurg.2025.1750529 (PMC12855474; doi:10.3389/fsurg.2025.1750529)
Supplement: Supplementary file 1 [file Table1.docx]

**Participants in standard development**

1. Bing Shi, The Eighth Medical Center of Chinese PLA General Hospital. 2. Qin Li, Aist Medical Beauty Group. 3. Shufan Wu, Zhejiang Provincial People's Hospital. 4. Jianhong Long, Xiangya Hospital of Central South University. 5. Ge Zhang, Zhengzhou Hualing Medical Beauty Hospital. 6. Hongmei Liu, Beijing Meiyan Medical Beauty Clinic. 7. Hongbin Xie, Peking University Third Hospital. 8. Yang Wang, Beijing Rende Yimei Medical Beauty Clinic. 9. Wei Xia, Xi'an Boshixiangban Medical Beauty Clinic. 10. Zongke Guo, Zhongda Hospital of Southeast University. 11. Wei Hong, Jingfu Medical Beauty. 12. Baohua Pan, Chongqing Huamei Plastic Surgery Hospital. 13. Sheng Han, Beijing Shouxi Lige Medical Beauty Clinic. 14. Zhongsheng Sun, Guangdong Second Provincial People's Hospital. 15. Yuanhong Li, Shenyang Yanyue Medical Beauty Clinic. 16. Xuefeng Han, Plastic Surgery Hospital of Chinese Academy of Medical Sciences. 17. Xiaoyang Wang, Shanghai Yanfan Medical Beauty Clinic. 18. Xiaochun Yu, Beijing Lido Medical Beauty Hospital. 19. Yang Gao, The Third Affiliated Hospital of Chongqing Medical University. 20. Dong Zeng, Guangzhou Zixin Plastic Surgery Hospital. 21. Wuyi Shen, The Third Affiliated Hospital of Zhejiang Chinese Medical University. 22. Yonggang Yu, Shenzhen Feifan Medical Beauty Hospital. 23. Pei Du, Shanghai Zhenjing Clinic. 24. Zhao He, Chengdu Jinmeishaohua Medical Beauty Clinic. 25. Yongjie Liu, Hangzhou Shiguang Medical Beauty Hospital. 26. Haibin Gu, Hangzhou Yueke Medical Beauty Clinic.27. Guoshou Jin, Kunshan Lirui Medical Beauty Clinic.28. Xiaoxu Li, Shandong Yimei (Tiancheng) Group. 29. Jianying Zhu, Shijiazhuang Yafangya Medical Beauty Hospital. 30. Guotian Liu, Xi'an Yixing Medical Beauty Hospital.

Five Medical Beauty Enterprises. Imeik Technology Development Co., Ltd. Johnson & Johnson (Shanghai) Medical Devices Co., Ltd. Shanghai Gaoshi Medical Technology Group Co., Ltd. Scienfield (Beijing) Trading Co., Ltd. Zhejiang Weidu Medical Devices Co., Ltd.
