# Supplementary figures and images for: Group standardization of Chinese experts specification of operating techniques for facial embedded thread lift
Source: Front Surg. 2026 Jan 16;12:1750529. doi: 10.3389/fsurg.2025.1750529 (PMC12855474; doi:10.3389/fsurg.2025.1750529)

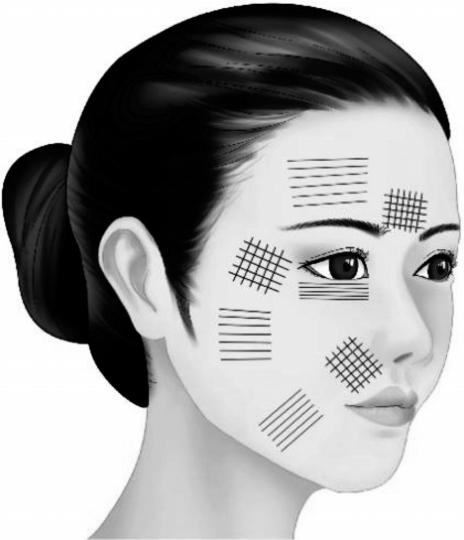

Supplement: Supplementary Figures S1 — Smooth thread and spiral thread can be applied to different designs according to different requirements. Application of crossroad net design can boost the volume. Combined application of spiral thread and barbed thread through vertical burying can assist the lifting effect of barbed thread. The spiral thread buries perpendicularly to the frontal muscle, platysma and other intramuscular burying can relax the muscle respectively. The intramuscular thread parallel to the orbicularis oculi muscle can enhance the muscular strength to improve the herniation of lower eyelid fat. [file Image1.jpeg]

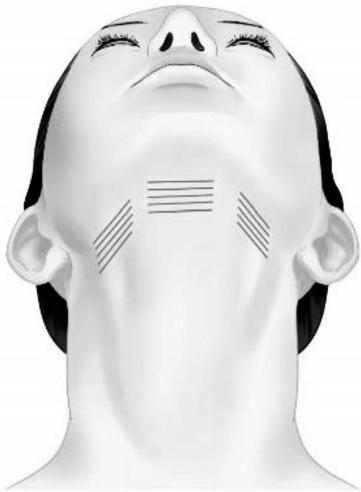

Supplement: Supplementary Figures S2 — The operation can also facilitate the improvement of neck lines as well as lifting of lower face. [file Image2.jpeg]
